# Using Early Change to Predict Outcome in Cognitive Behaviour Therapy: Exploring Timeframe, Calculation Method, and Differences of Disorder-Specific versus General Measures

**DOI:** 10.1371/journal.pone.0100614

**Published:** 2014-06-24

**Authors:** Peter Schibbye, Ata Ghaderi, Brjánn Ljótsson, Erik Hedman, Nils Lindefors, Christian Rück, Viktor Kaldo

**Affiliations:** 1 Department of Clinical Neuroscience, Division of Psychiatry, Karolinska Institutet, Stockholm, Sweden; 2 Section of Psychiatry, Sundsvall Härnösand County Hospital, Västernorrland County Council, Sweden; 3 Department of Clinical Neuroscience, Division of Psychology, Karolinska Institutet, Stockholm, Sweden; 4 Department of Clinical Neuroscience, Osher Center for Integrative Medicine, Karolinska Institutet, Stockholm, Sweden; Banner Alzheimer's Institute, United States of America

## Abstract

Early change can predict outcome of psychological treatment, especially in cognitive behavior therapy. However, the optimal operationalization of “early change” for maximizing its predictive ability, and differences in predictive ability of disorder-specific versus general mental health measures has yet to be clarified. This study aimed to investigate how well early change predicted outcome depending on the week it was measured, the calculation method (regression slope or simple subtraction), the type of measures used, and the target disorder. During 10–15 weeks of internet-based cognitive behavior therapy for depression, social anxiety disorder, or panic disorder, weekly ratings were collected through both disorder-specific measures and general measures (Outcome Questionnaire-45 (OQ-45) and Clinical Outcomes in Routine Evaluation-10 (CORE-10)). With outcome defined as the disorder-specific measure, change at week four was the optimal predictor. Slope and subtraction methods performed equally well. The OQ-45 explained 18% of outcome for depression, 14% for social anxiety disorder, and 0% for panic disorder. Corresponding values for CORE-10 were 23%, 29%, and 25%. Specific measures explained 41%, 43%, and 34% respectively: this exceeded the ability of general measures also when they predicted themselves. We conclude that a simple calculation method with a disorder-specific measure at week four seems to provide a good choice for predicting outcome in time-limited cognitive behavior therapy.

## Introduction

Cognitive behaviour therapy (CBT) has a large empirical base supporting its efficacy in the treatment of panic disorder, social anxiety disorder, and depression [1]–[3]. Nevertheless, about a third of patients do not sufficiently respond to CBT [4]–[7] and in clinical settings a small number of patients (5–10%) deteriorate during treatment [8]–[10]. There is a need to investigate clinically useful methods and instruments for early prediction of treatment outcome. Even though it would be desirable if patient characteristics could prospectively predict whether a treatment will be successful or not, prediction studies of CBT for panic disorder, social anxiety disorder, and depression have failed to identify stable pre-treatment patient characteristics that reliably predict treatment outcome with the exception of baseline symptom severity [11], [12].

There is an emerging body of knowledge suggesting that early improvement, i.e. symptom reduction in the initial phase of treatment, is strongly related to treatment outcome in CBT for anxiety and depression [13] and that formal assessment of improvement in general are better predictors than therapists' clinical judgement [14]–[17]. However, there is substantial heterogeneity across studies in terms of how early improvement is defined and between which time points during therapy it is assessed [18]–[20]. No single time point or number of weeks in therapy has emerged as the most optimal for measuring early change. As for definition of early improvement, the studies cited above used different complex and power demanding statistical methods to predict treatment outcome. In many cases, individual slope patterns are used to assess early improvement. Such complex methods may limit the implementation of early improvement assessment. If the clinician is required to use statistical software to determine whether a patient has made an early improvement, this important information is less likely to be available in routine clinical practice. Consequently, the core concern of the present study was to identify the most optimal and simple to use methods and instruments for early prediction of treatment outcome.

Since measures of depressive symptoms and anxiety are highly correlated, a general measure including common aspects of symptoms and functioning could be useful and have some advantages over using several different specific measures [21]. In the present study, we use the term general measure when referring to measures that are designed to be suitable for administration for several psychiatric disorders and assess at least two symptom domains. Two of the most widely used general measures are the Outcome Questionnaire – 45 (OQ-45) [2] and the Clinical Outcomes in Routine Evaluation – 10 (CORE-10) [22]). Hannan et al. [14] have shown that the predictive power of OQ-45 is superior to assessment based on general clinical experience of the therapists. Furthermore, providing therapists with the results of OQ-45 before the start of each session has been shown to significantly improve outcomes in psychological treatment [9], [23]–[25]. However, no studies have compared how well change during therapy measured with general measures and disorder-specific measures correlate to each other and to what degree each kind of measure predict outcome. Such a comparison is essential before making an informed decision on the use of either general or specific measures.

One aspect to consider when administering symptom measures frequently during therapy is the length of the instruments. Lengthy and detailed measures might lead to reduced patient adherence when used in continuous evaluation. However, in order for shorter general measures, such as the CORE-10, to be clinically relevant their predictive power needs to be as good as that of more comprehensive general measures. As far as we know, no comparisons between shorter and longer questionnaires have been made before in this context and it would thus be important to compare the predictive powers of the OQ-45 with the shorter CORE-10.

In the recent decade, internet-based Cognitive Behavior Therapy (ICBT) has emerged as an effective treatment alternative for anxiety disorders and depression [26]. ICBT is based on the same treatment methods as face-to-face CBT and could be described as clinician-guided online bibliotherapy with therapist support through email [27]. Although the processes of change may be slightly different in face-to-face and Internet-based psychotherapy, the latter provides a good opportunity for investigating the questions raised above. ICBT has been evaluated for at least 25 psychiatric and functional disorders in more than 100 randomized controlled trials (RCTs) [26]. ICBT for panic disorder, social anxiety disorder, and depression present treatment effects equal to face-to-face CBT [28]–[30]. Studies investigating predictors in ICBT and face-to face CBT for these three disorders have generally failed to find stable moderators, potentially suggesting that therapeutic processes are similar in ICBT and face-to-face CBT [31]–[33]. To our knowledge, no prior study has investigated the predictive power of early improvement in ICBT.

There were two specific aims of the present study. First, we wanted to explore to what extent early change predicts outcome depending on:

(1a) the method that was used to calculate early change,

(1b) the week at which early change was measured, and

(1c) whether the preferred calculation method and time-point for defining early change differed between general and disorder-specific measures.

Second, we wanted to explore:

(2a) how well change during therapy on two general measures (OQ-45 and CORE-10) and disorder-specific measures correlated with each other, and

(2b) whether their ability to predict outcome of ICBT differed.

## Materials and Methods

### Participants and the treatment context

Patients were recruited from the Internet Psychiatry Clinic (for a detailed description of the clinic, see Hedman et al., 2013) in Stockholm, Sweden. The unit has been providing ICBT as part of their routine care since 2007 and the service is available through referral and self-referral to all adults in Stockholm County. ICBT has previously shown good results also in routine care [34], [35]. Patients were diagnosed by a psychiatrist or a resident physician under supervision through the Mini-International Neuropsychiatric Interview (MINI) [36]. At the time of recruitment, the clinic offered diagnosis specific ICBT for patients with panic disorder, social anxiety disorder, and unipolar major depression, i.e. the clinic had specific treatment protocols for each of the three psychiatric disorders. The main inclusion criterion was that participants should have a principal diagnosis of panic disorder, social anxiety disorder or depression. Co-morbid psychiatric diagnoses were allowed as long as they were considered secondary. Patients were excluded if judged unsuitable for ICBT due to suicidal ideation, comorbid psychiatric disorders, or substance abuse were referred to other health care providers and did not receive ICBT. During the recruitment phase, all patients who underwent an online pre-interview assessment at the clinic were given the opportunity to participate in this study. Patients were informed that participation in this study meant completing two weekly questionnaires in addition to the weekly measures regularly used at the clinic, and that the decision to participate or refrain from participation in the study would not affect any aspects of their care at the clinic. Patients provided informed consent to participate in the study during the pre-interview online assessment.

**Figure 1:**
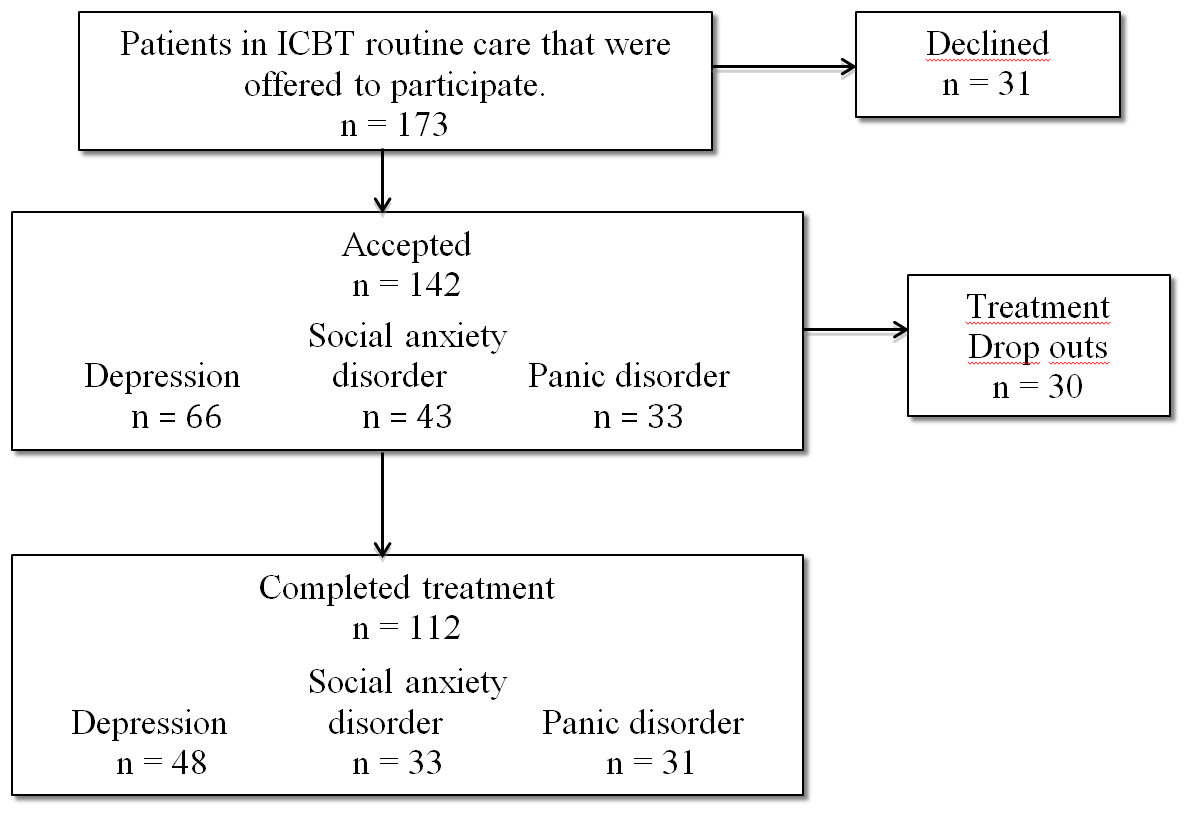
Flow of participants throughout the study.

The characteristics of the patients are presented in Table 1 and the inclusion and dropout is illustrated in Figure 1.

**Table 1:** Sample characteristics.

	Depression n = 66	Panic disorder n = 33	Social anxiety disorder n = 43	All participants N = 142
Average age	38.11	35.61	37.28	37.27^a^
Sex (% women)	68%	79%	56%	67%
Married/cohabiting	48%	70%	44%	52%
Working	77%	79%	61%	73%
Education:				
Elementary school	0%	3%	2%	1%
High school	30%	39%	26%	31%
University/College	70%	58%	72%	68%

a Age range was 19 to 73 years.

### Ethics statement

The study was approved by the regional ethical review board in Stockholm (2009/1809-31/3).

### Questionnaires

#### The Clinical Outcomes in Routine Evaluation–10 (CORE-10)

CORE-10 is a self-assessment questionnaire with 10 items chosen from the Clinical Outcomes in Routine Evaluation – Outcome Measure (CORE-OM), which is a general measure with 34 items developed to assess global distress in a broad range of patients. The two measures are highly correlated and the internal consistency and concurrent validity of CORE-10 is considered good [22]. The Swedish version of CORE-OM has good test-retest reliability, good homogeneity and the same factor structure as the original version (Unpublished data). The items for CORE-10 used in this study were extracted from the translated version.

#### Outcome Questionnaire-45 (OQ-45)

The OQ-45, consisting of 45 items, is constructed as a general measure for use by all patients in health care, regardless of psychiatric diagnosis. The questionnaire is divided into three subscales: psychological symptoms, interpersonal problems, and functioning in social roles. The OQ-45 has good reliability, high test-retest reliability, and good concurrent validity [15], [37] The predictive power of the OQ-45 on reliable deterioration has been evaluated [15], [38], [39]. These studies show a hit rate of 80% (i.e., the percentage of patients being correctly categorised), a sensitivity of 88% (the proportion of those who actually deteriorated and was correctly categorized), and a positive predictive value of 20%, representing the proportion of patients who were predicted as deteriorating and who had actually deteriorated by the end of the therapy. For the translation to Swedish, and for the back-translation, three independent translators were used, and the Swedish version has presented good internal consistency [40].

#### Disorder-specific measures

Three disorder specific measures were used, one in each treatment. Participants who received ICBT for depression were assessed with the Montgomery Åsberg Depression Rating Scale –Self rated (MADRS-S), which consists of nine items (scale range = 0–54) and has good convergent validity, test-retest reliability, and internal consistency [41], [42]. A cut-off score of 12 on the MADRS-S has been suggested for discriminating between patients with depression [43].

The Panic Disorder Severity Scale – Self-Report (PDSS-SR) is a seven item scale (scale range = 0–21) that was used to measure panic-related anxiety among participants who underwent ICBT for panic disorder [44]. The PDSS-SR has shown good convergent validity, test-retest reliability, internal consistency, sensitivity to change and some discriminative validity [45], [46]. Although there are no established cutoffs for the clinical range in terms on the PDSS-SR, reports on the similar clinician administered version of the same scale, suggest that a score of 6 discriminates between patients with and without panic disorder [47].

The Liebowitz Social Anxiety Scale – Self-Report (LSAS-SR) is a scale with 24 items rated on two dimensions, anxiety and avoidance (scale range 0–144). The LSAS-SR was used to measure social anxiety in participants receiving ICBT for social anxiety disorder [48]. The LSAS-SR has good convergent validity, test-retest reliability, internal consistency, and discriminative validity [49]. A cutoff score of 30 has been shown to be an optimal cutoff for identifying persons with social anxiety disorder, and a score of 60 discriminates between generalized and non-generalized social anxiety disorder [50].

### Treatments

The patients went through ICBT programs tailored for their specific principal diagnosis, i.e., depression, social anxiety disorder or panic disorder. Thus, each participant could access only one of the three ICBT programs available at the clinic. The general principle of the treatment was that patients should be exposed to the same interventions as in conventional CBT. Throughout the treatment, all patients had access to an online therapist who supervised the progress and provided individual feedback on homework exercises. Each program was divided into several modules and upon module completion, therapists granted access to the next module. Patients could send messages to their therapist at any time and expect a reply during the next weekday. In general, there was no face-to-face contact between therapist and patient; however, if the therapist deemed it necessary they could contact the patient by telephone. In addition, patients had access to an online discussion forum where they could communicate anonymously with each other. The expected duration of treatment was 10 weeks in ICBT for depression and panic disorder, and 15 weeks in ICBT for social anxiety disorder. The treatment could in rare cases be prolonged if the therapist considered that the patient would benefit from it. All online therapists were psychologists, licensed or resident, with thorough training in CBT. All treatment protocols had previously demonstrated efficacy in randomized controlled trials of ICBT [28]–[30] and were based on the principles of recognized cognitive behavioural treatments for the specific diagnoses [51]–[54]. Large scale effectiveness studies of the treatments have demonstrated that the treatments yield large effect sizes on measures of panic-related anxiety, depressive symptoms and social anxiety [35], [55].

### Procedure

The disorder-specific measures (MADRS-S, PDSS-SR, and LSAS-SR) and the two general measures (OQ-45 and CORE-10) were administered after each week in treatment. This meant that participants in treatment of panic disorder and depression completed the questionnaires 10 times, while those in ICBT for social anxiety disorder completed the assessments 15 times. The questionnaires appeared immediately when the patient logged in and had to be completed before the patient could access the treatment. Any missing weekly questionnaires were replaced with the questionnaires from the previous week. After four weeks of treatment patients were asked how they experienced answering the weekly questionnaires. If a patient reported that completing the extra study questionnaires was in any way preventing his or her engagement in treatment the CORE-10 and OQ-45 measures were removed from the weekly measures. Four cases were excluded this way and were considered dropouts in the data-analyses. Although therapists could access the summary scores from the questionnaire during treatment, they were not actively supplied with the scores in order to monitor treatment progress.

### Definition of early change and outcome

To determine the week best suited for measuring early change, the measures' predictive ability in each week must be weighed against the need to make a prediction as early as possible. Change during the first 5 weeks was considered early since it represents the first half of the treatment. The clinical usefulness of predictions of outcome is considered to decrease closer to the end of therapy when there is less room for change and more time has been spent in an ineffective treatment.

Two methods were used to calculate early change (research question 1a):

1. *Subtraction*: Pre-treatment rating minus the target weekly rating. The values of weekly ratings between these two ratings were ignored.
2. *Slope*: For each patient, individual slope estimates from the pre-treatment to the target weekly rating were calculated through regression analysis with week as independent variable and weekly ratings as the dependent variable. The regression coefficient (the slope of the regression line) was used as the individual predictor. Thus, in the Slope method, all ratings up to the target week influence the predictor value.

For explorative purposes, non-linear predictions were also performed, by stepwise adding the square and cube level of the predictor to the regression models used for testing the subtraction method.

Outcome was defined as the difference between pre-treatment and post-treatment values on the relevant disorder-specific questionnaire. In the comparisons between disorder-specific and general measures in research question 2b, the latter were also used to define outcome. If the post-measure was missing the last completed assessment at week 7 or later was used as instead. In the social anxiety disorder treatment, completed assessments after week 12 replaced missing post-measures, since this treatment was 15 weeks long. Patients missing both post-treatment data and or weekly ratings after week 7 (or week 12 for social anxiety disorder), were considered dropouts and not included in the analyses.

### Statistical analyses

Predictive ability was defined as the amount of variance in the outcome (dependent variable) that could be explained by early change (independent variable, calculated according to either the *subtraction* or *slope* method). Explained variance in outcome was obtained by linear regressions where the regression coefficients (r^2^) were transformed to percentages and presented on graphs. Confidence intervals (95%) were calculated for the r^2^ values. Regression analysis was used to control if baseline symptom severity was a better predictor then early change and if it decreased the amount of variance explained by early change. The square and cube of the predictor in the subtraction method was added stepwise to explore if non-linear models would strengthen the predictive capacity. Before the regressions were carried out, these missing data was imputed with the Last-Observation-Carried-Forward principle.

Data were explored for outlier and extreme values and significant skewness and curtosis: extreme values were replaced with the nearest value within three standard deviations of the mean. The distribution of data was considered sufficient for parametric analyses and transformation was not needed. Within each questionnaire no questions went unanswered, as incomplete responses to questionnaires could not be submitted. For the statistical analyses SPSS 19 was used.

## Results

### Attrition

Missing data during the first five weeks was 8%. Six patients discontinued the use of the general questionnaires, of which four patients did so before week 7, and they were therefore considered dropouts due to lack of outcome data.

### The overall effect of ICBT

**Figure 2:**
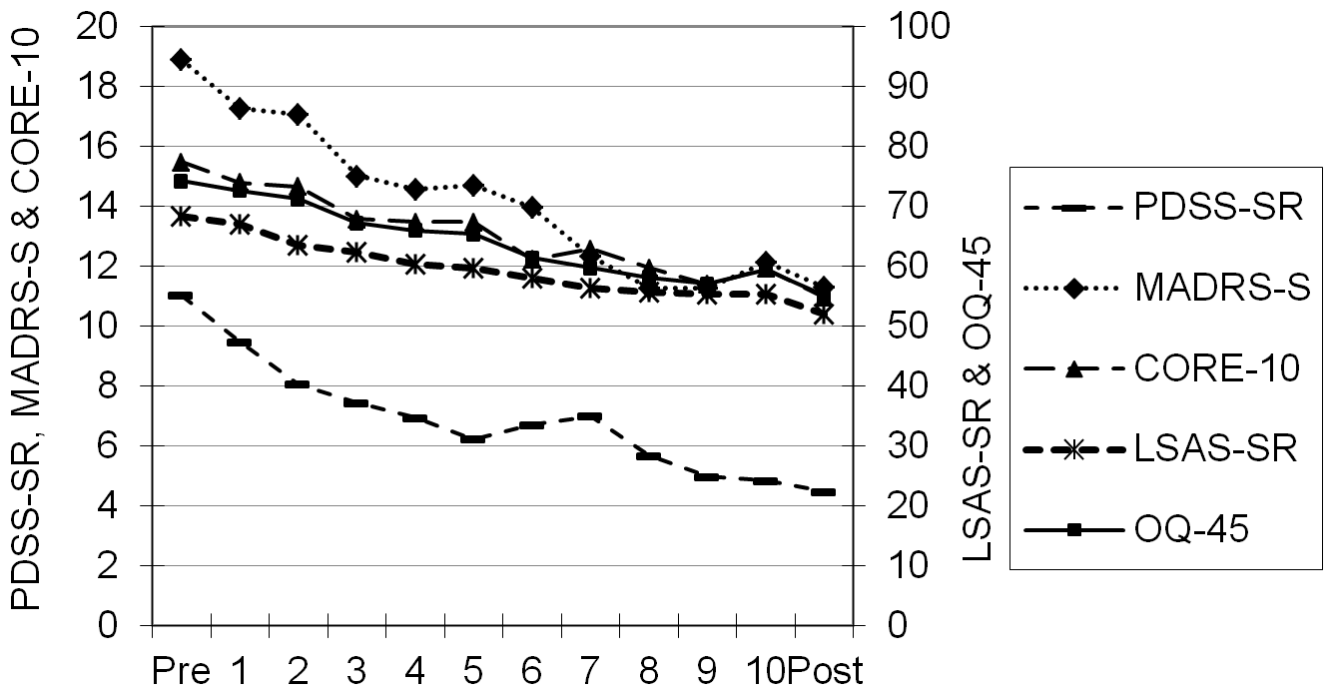
Mean value at each week for all measures and all patient groups.

Weekly changes in the average scores of both specific and general measures are presented in Figure 2.

An expected decrease in symptoms based on both general and specific measures was seen from pre- to post-treatment (Figure 2). For the general measures, across all patients, the mean effect size was 0.73 for Core-10 and 0.78 for OQ-45 indicating a moderate to large effect. The complete evaluation of the ICBT conducted at the Internet psychiatry unit is beyond the scope of this paper, but has so far been presented in two studies [35], [55] with positive results. Among patients receiving ICBT for panic disorder 32 (97%) had a baseline score on the PDSS-SR above the clinical cutoff, i.e. 6 or higher, while 10 (31%) patients scored in the clinical range at post-treatment. In the sample with social anxiety disorder, 44 (98%) patients scored above the suggested clinical cutoff, i.e. 30 or higher on the LSAS-SR, at baseline. At post-treatment 38 (88%) scored in the clinical range on the LSAS-SR. In the group of patients receiving ICBT for depression 60 (90%) had a MADRS-S score indicating depression, i.e. 12 or higher, at baseline while 37 (55%) scored in the clinical range at post-treatment.

### (1a) Which method (Subtraction or Slope) for calculating early change was the best predictor of outcome?

**Figure 3:**
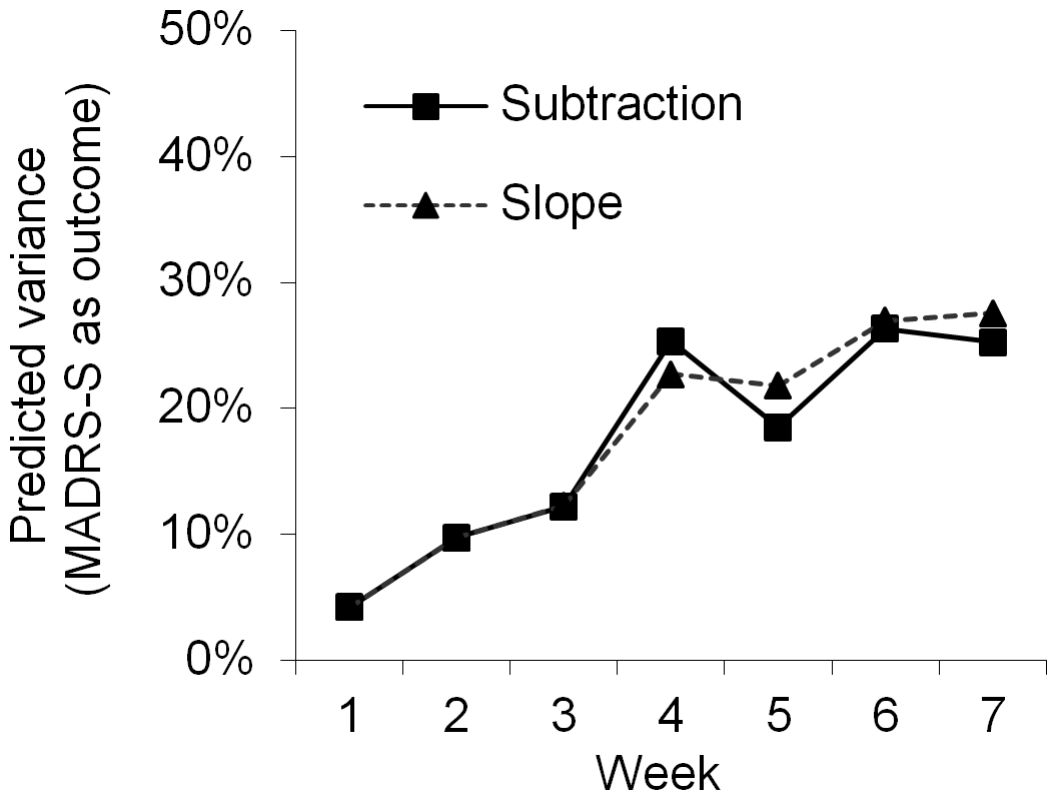
Explained variance in outcome on specific measures for all patients and questionnaires using two different statistical methods.

The pre- to post treatment change on the appropriate disorder-specific measure was used as outcome. For each diagnostic group, the explained variance in outcome was calculated through separate analysis with OQ-45, CORE-10, or the disorder-specific measure as indices of early change. This procedure resulted in nine values for the explained variance for Subtraction versus Slope method at each week. The average of these nine values is presented in Figure 3 for each calculation method.

The difference in amount of explained variance between the methods did not exceed 4% on any assessment point from week 1 to week 5 and none of the methods was consistently superior to the other in terms of prediction during the early phase of the treatment. Sub analyses for each diagnostic group and measure also rendered similar results. As no method could be established as superior and in order to limit the size and complexity of the result section the subtraction method, which is the simpler method, was used to investigate the remaining research questions.

The possible additional effects of non-linear models were explored by stepwise adding the square and the cube of the difference between the pre-value and the value of the current week (i.e. subtraction method). However, in only 5.9% of the cases (no corrections for multiple testing applied) this resulted in significantly more variance being predicted and the maximum gain in explained variance was 8.8%. This led to the decision of not using non-linear prediction models in the following analyses.

### (1b) At which point in treatment is early change best measured to predict treatment outcome?

**Figure 4:**
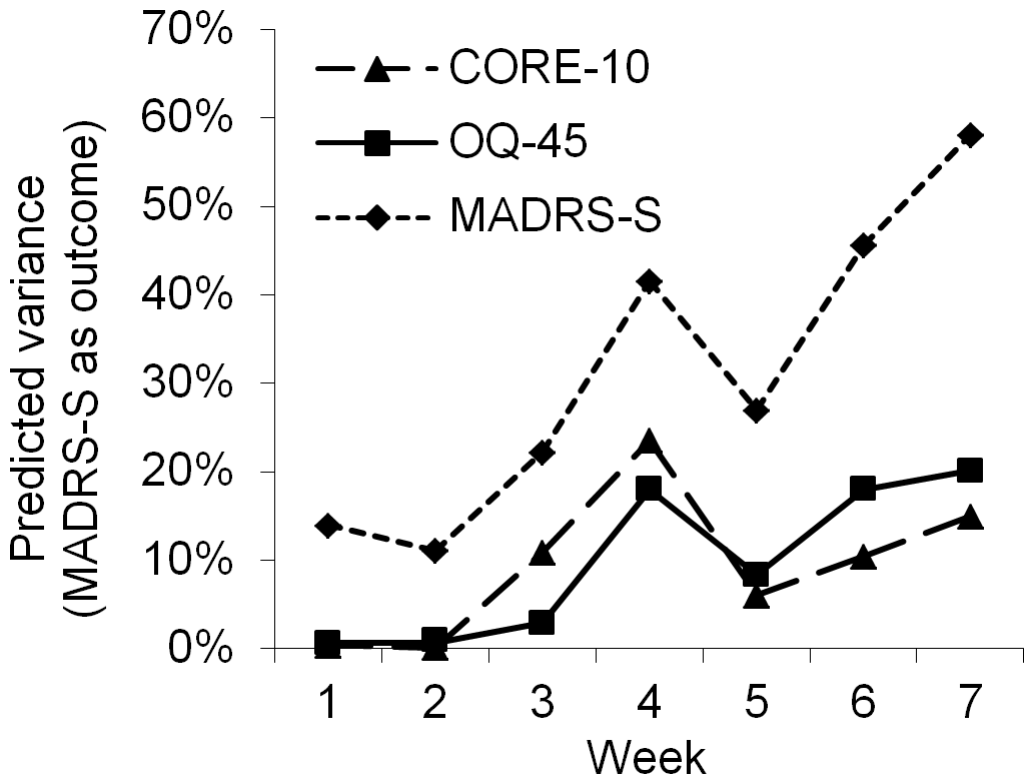
Explained variance in outcome of MADRS-S predicted by early change among patients with depression.

**Figure 5:**
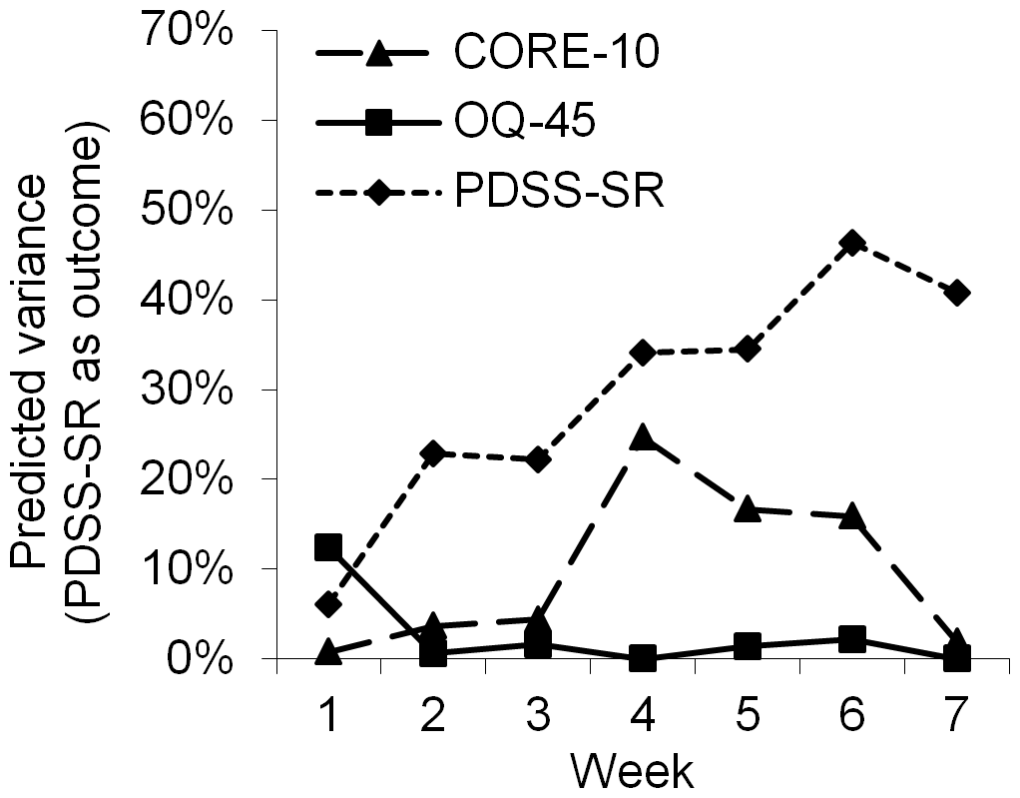
Explained variance in outcome of PDSS-SR predicted by early change among patients with panic disorder.

**Figure 6:**
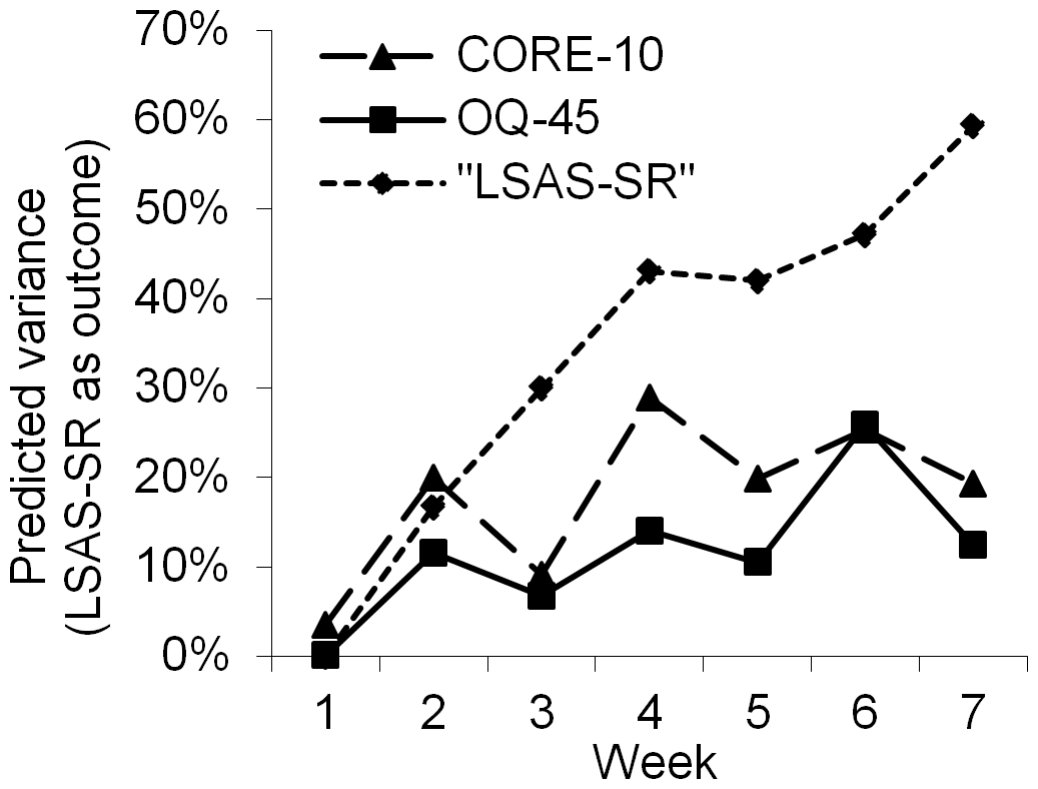
Explained variance in outcome of LSAS-SR predicted by early change among patients with social anxiety disorder.

**Figure 7:**
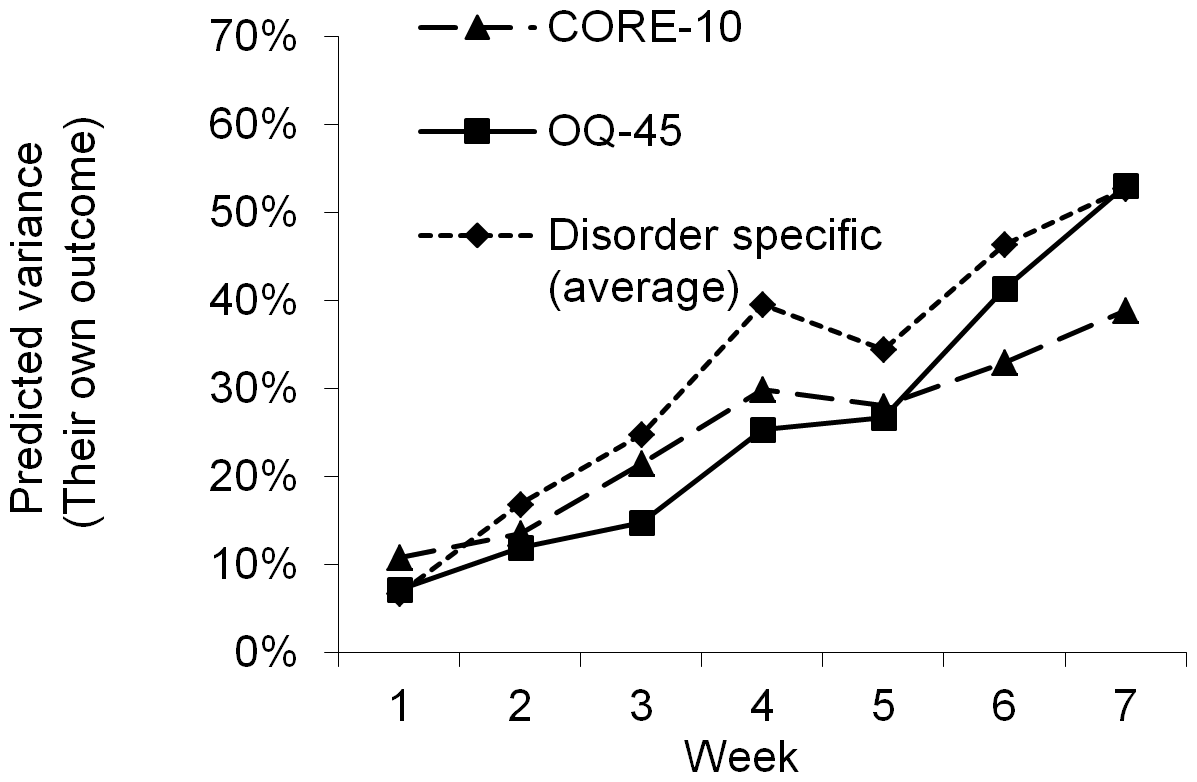
Explained variance when each measure predicts itself (presenting the average predictive ability for all three specific measures).

The amount of explained variance changed over the weeks as function of different combinations of predictors and outcome measures (Figures 3–7). The extent (on average) to which all measures predicted outcome (disorder-specific measures) is presented in Figure 3. As shown in Figure 3, there was a strong association between time and predictive power from baseline to week 4. A similar pattern was observed when the predictive ability of the different measures was analyzed separately for each diagnostic group (Figures 4–6), and where the general measures predict themselves as outcome (Figure 7). When each measure predicts itself (Figure 7) a general decrease in explained variance is seen between week 4 and 5, which is then followed by a slight increase. This increase beyond week 5 is less evident when general measures are used to predict disorder specific measures. When the need to predict as much of the outcome as possible was balanced against the importance of making an early prediction, prediction at the fourth week of treatment was judged to be the best option. We could not find any appropriate statistical method to weigh both the time of prediction and strength of prediction into the same significance test. Week 4 was thus chosen not because of it having a statistically significant advantage over week 5 but because no difference to week 5's advantage was found in the results (week 4 and 5 showing overlapping confidence intervals) and week 4 has the advantage of providing earlier identification.

### (1c) Does the calculation method and preferred week used to define early change differ between general and disorder-specific measures?

Sub analyses of the effects of the calculation method on the general and specific measures did not reveal any differences. A visual inspection of Figure 3–7 still supported week 4 as the best week for measuring early change, regardless of type of measure and disorder.

### (2a) How well do change on the two general measures (OQ-45 and CORE-10) and the disorder-specific measures correlate with each other?

Change score correlations between general and disorder specific measures are presented in Table 2. The OQ-45 and the CORE-10 were highly correlated with MADRS-S, moderately correlated with LSAS-SR, and slightly correlated with PDSS-SR.

**Table 2:** Correlation between change from pre- to post-treatment.

	OQ-45	CORE-10
MADRS-S (N = 48)	.71**	.69**
LSAS-SR (N = 33)	.54**	.57**
PDSS-SR (N = 31)	.31	.36*

*p<.05, **p<.01.

The general measures were highly correlated with each other (*r* = .82; *p*<.001).

### (2b) Do the two general measures (OQ-45 and CORE-10) and the disorder-specific measures differ in their ability to predict outcome?

When the outcome was defined as the specific measure, the specific measures for depression (MADRS-S), panic disorder (PDSS-SR) and social anxiety disorder (LSAS-SR) were superior to the general measures for predicting outcome (Figure 4,5 and 6). For depressed patients (Figure 4), all predictions with MADRS-S were statistically significant, whereas, predictions at weeks 1, 2 and 3 for OQ-45 and weeks 1, 2 and 5 for CORE-10 were not statistically significant. All r^2^ values had overlapping confidence intervals except when comparing MADRS-S week 7 to CORE-10 week 7. For patients with panic disorder (Figure 5), predictions with PDSS-SR were significant at weeks 2–7, while the CORE-10 significantly predicted outcome at weeks 4, 5 and 6. The OQ-45 had no significant predictive effect on any of the assessment points. The only confidence intervals for r^2^ values that did not overlap were week 4 and 7 for OQ-45 and PDSS-SR. A similar pattern was shown among patients who received treatment for social anxiety disorder (Figure 6). The LSAS-SR significantly predicted outcome at weeks 2–7, whereas the CORE-10 was significantly associated with outcome at weeks 2 and 4–7. The OQ-45 significantly predicted outcome at week 4, 6 and 7. All the r^2^ values had overlapping confidence intervals except for week 7 for PDSS-SR and OQ-45. To increase readability, the r^2^ values are not presented in the text or in the Figures, but they can be obtained from the corresponding author.

In all analyses above, specific measures were used to define outcome, which might create some bias, favoring the specific measures since they predict themselves. To control for this bias, a new analysis where the OQ-45 and CORE-10 instead predicted themselves was performed. The general measures then approached but still did not attain similar levels of predictive capacity (but showed overlapping confidence intervals) as when specific measures were used. This becomes clear when Figure 7 is inspected, showing the two general measures predicting themselves, together with the average predictive ability for the disorder specific measures predicting themselves. The predictions by OQ-45 and the CORE-10 in Figure 7 were statistically significant, partly due to higher power as all patients were included in these analyses; however, there were no large or significant differences between the OQ-45 and the CORE-10 in these predictions.

As a final comparison, the pre- to post changes in the general measures were used as the outcome and the specific measures as predictors. Overall, this demonstrated weak predictive abilities except for MADRS-S, which significantly predicted change in the general outcome measures. Using OQ-45 as outcome, early change (i.e. change from pre-treatment to week 4) in MADRS-S predicted 23% of the outcome among patients who received ICBT for depression, LSAS-SR predicted 4% of the outcome in ICBT for social anxiety disorder and the PDSS-SR 0% in the cohort of participants that underwent ICBT for panic disorder. The corresponding figures for explained variance with CORE-10 as the outcome, early prediction was 34% with MADRS-S, 6% with LSAS-SR and 2% with PDSS-SR.

Since week 4 presents as the most promising week to measure early change, a more detailed view of the predictions made at this week is presented in Table 3, in which it is shown that specific measures predicted a larger proportion of the variance in outcome than the general measures.

**Table 3:** Explained variance (r^2^) in outcome on disorder specific measures as predicted by general and specific measures at week 4.

Patient group	OQ-45	CORE-10	Specific measure^a^
Panic disorder	.00	.25**	.34**
Depression	.18**	.24**	.41**
Social anxiety disorder	.14*	.29**	.43**

*Note.* df range between 30 and 47.

a PDSS-SR for panic disorder, MADRS-S for depression and LSAS-SR for Social anxiety disorder.

*p<.05, **p<.01.

Baseline severity may be associated with early improvement and final outcome. Therefore, baseline severity was controlled for in the model by entering it in the first block, to investigate whether early change (difference from start to week 4) could significantly predict a meaningful portion of the variance in outcome. These analyses were made for each measure separately and showed that in all cases early change was still a significant predictor, explaining major part of the variance in outcome. As an example, early change in LSAS explained 43.1% of the variance in total change in LSAS. After entering LSAS baseline in first block, the total amount of explained variance increased to 44.5%. Baseline value of LSAS was not a significant predictor, but the early change was a significant contributor. As an example of general measures, early change on Core-10 explained 29.9% of variance in total change in Core-10 (from baseline to post). When baseline value of Core-10 was entered in the model, the total amount of explained variance increased to 31.3%. The baseline value of Core-10 was a significant predictor explaining 8.1% of the variance, and early change still significantly explained another 23.2% of the variance. Similar patterns were observed when general measures were predicted by specific measure and vice versa.

Explained variance was also calculated for each group of patients using the respective general measure as both predictor (week 4) and outcome. For patients with depression, the OQ-45 explained a significant portion of outcome (r^2^ = .34, *p*<.001), as did the CORE-10 (r^2^ = .49, *p*<.001). For patients with panic disorder, the corresponding figures was r^2^  = .13 (*p* = .043) with OQ-45 and r^2^ = .16 (*p* = .025) with CORE-10. For social anxiety disorder, the explained variance was r^2^ = .19 (*p*<.011) with OQ-45 and r^2^ = .21 (*p* = .007) with CORE-10.

## Discussion

The general aim of the present study was to investigate clinically relevant aspects of early improvement in CBT delivered via the Internet for panic disorder, social anxiety disorder and depression. More specifically, we explored differences in predictive power between general measures (OQ-45 and CORE-10) and disorder specific instruments (PDSS-SR, LSAS-SR and MADRS-S), and how different factors, such as various methods of calculating early change and a time-frame for defining early change, influenced these predictions.

In terms of different methods for calculating early change, the results showed that neither subtraction nor slope could be established as superior, and that the addition of non-linear prediction models in the vast majority of analyses did not significantly enhance the predictive ability. This is considered clinically important as the subtraction method is straightforward and easy to use in any clinical context. These findings are in accordance with the work by Percevic et al. [38], showing that the use of multiple assessment points does not improve prediction compared to using just two assessment points.

In the investigation of the most optimal time point during the early phase of therapy for prediction of treatment outcome there was a general tendency across different questionnaires and treatment groups. The extension of the period for defining early change from pre-treatment to the initial weeks of the therapy up to week 4 resulted in a steady increase in explained variance; however, between week 4 and 5 this trend changed. This decline in predictive ability is difficult to explain. It is most apparent in the treatment of depression and for the general measures in the other treatments. Thorough investigations of the data did not reveal any error that could explain this finding. Interestingly though, Figure 2 shows a temporary halt in distress reduction between week four and five for MADRS-S, OQ-45, and CORE-10 but not for the other measures. Analyses of all measures showed that the explained variance continued to increase after week five if the measures predicted their own outcome but not as clearly when the general measures predicted disorder specific outcome (figure 3–5). This indicating that using later time-points when predicting other aspects of outcome is not always an advantage. Taken together, if a single point in time during treatment is to be chosen, week four seems to be a suitable week for early prediction of outcome. A detailed discussion of what should be done if a negative outcome is expected is beyond the scope of this paper, but it can be worth mentioning that the feedback of continuous evaluation without further instructions has been shown to have a positive effect on treatment results [24]. This suggests that clinicians perform effective changes in their therapy when negative feedback is presented even without instructions. The effect of a clinical problem solving tool to identify what should be changed in therapies that are not on track has been examined and shows promising results [9], [56]. In ICBT the clinician is faced with decisions of whether changing to regular CBT, increasing support, e.g. through telephone contact, or other options, such as initiating pharmacological treatment.

The general and disorder-specific measures were compared to determine how well they predicted outcome of therapy. For all treatment groups the specific measures explained an equal or higher portion of variance in outcome than the general measures. Although confidence intervals overlapped to a high extent, the results indicated that specific measures were more suitable for continuous evaluation of treatments when the outcome is based on specific measures. Although the results might, to some extent, be a consequence of the same instrument being used to predict itself (i.e., an instrumental confounding), when the general measures were used to predict themselves, they still did not reach the same level of predictive ability as when the disorder-specific measures predicted themselves (but showed overlapping confidence intervals). Since specific measures showed a much lower ability to predict the outcome when it was measured as pre-post change in general measures it is very important to consider how the outcome of therapy is operationalized and measured in order to be able to choose appropriate measure for continuous evaluation. If a change in general mental health is the primary goal of treatment, general measures should be considered for continuous evaluation while specific measures should be considered if disorder specific symptom reduction is the primary goal. This is an important aspect of the results showing that change in measures of general mental health does not always correspond to change in disorder specific symptoms. Thus indicating that they measure different constructs on which change is not always correlated.

CORE-10 and OQ-45 appeared equally effective for continuous evaluation and prediction of outcome, and pre-post measures correlated highly with each other, suggesting that they measure a similar construct.

There were differences in the general instruments predictive ability across the different patient groups. General measures predicted little variance in outcome for patients with panic disorder. This indicates that it is important to establish the validity of general measures for each patient group on which it is used. We also found that among the groups that were treated for panic disorder and social anxiety disorder, the general measures were weakly correlated even with themselves as outcomes. That is, improvement scores on OQ-45 and CORE-10 early in therapy had little predictive value of total improvement also when the scales predicted themselves. The low correlation in pre-post change between the specific measure for panic disorder and the general measures also indicated general measures might not work as well as outcome measures for patients with panic disorder as they do for patients with social anxiety disorder and depression. This suggests general measures for some disorders should be considered as a complement to rather than a replacement of disorder-specific measures. This contrasted to Lambert [21],who argues the correlation between instruments measuring anxiety and depression suggests the use of more general measures such as OQ-45 or CORE-10 for continuous evaluation.

If choosing among the general measures OQ-45 and CORE-10, the latter can be recommended as the two measures performed equally well, but CORE-10 has the clinical advantage of being shorter and thus demands less time for filling out and for scoring. It should however be noted that this decision is based on the measures predictive abilities, it is possible that the measures could provide the clinician with other meaningful information, which should also be taken into account when choosing measures. For example, the OQ-45 does contain questions on work impairment and substance abuse, which for some patients might be very relevant to continuously evaluate throughout treatment. Also, as the clinical usefulness of general measures seems to be moderated by type of psychiatric disorder and therefore it is recommended that the decision to evaluate treatments with the OQ-45 or the CORE-10 should be made separately for each psychiatric disorder.

There were some limitations to this study. The research was performed on time-limited, guided Internet-based CBT. Similar and extended research on face-to-face therapy and psychotherapies with orientations other than CBT is needed to investigate the applicability and generalizability of the findings. It is possible that early change better predicts outcome in therapies with standardized treatment length, than in therapies that are more flexible and where therapy length is increased for patients who experience little or no initial improvement [38]. Also, a low number of patients in some group comparisons resulted in relatively low statistical power. Finally, as no automatic feedback was used, but the data from the weekly ratings were available to the therapists, this might have affected the relationship between early change and outcome. However, the risk of such a confounding was low as ICBT in general, and the treatments in the present study in particular, are strictly manualized, which leaves less room for changes in therapeutic strategy.

Due to difference in statistical methods and design, the findings could not be easily compared to previous studies on the predictive quality of general measures during continuous evaluation. It should also be pointed out that it is the outcome and not the remaining change that was predicted. It is thus possible that remaining change tends to be independent of early change as indicated by the research of Percevic et. al. [38].

However, as the aim of this study primarily was to investigate the effect of using different assessment points, assessment methods, and methods of analysis in the prediction of outcome using early improvement within a clinically relevant context, we regarded such analyses to be beyond the scope of this paper.

To conclude, we view the findings of the present study as important as they provide new knowledge on clinically relevant aspects of early improvement in the treatment of panic disorder, social anxiety disorder and depression. The study provides support for the use of simple statistical methods to predict outcome and suggests week four as a suitable candidate for early prediction of outcome. The findings also demonstrate that if disorder specific symptoms are considered the primary outcome then disorder specific measures should be used as predictors rather than general measures. More research is needed on general measures to further investigate for which disorders they are most suitable for predicting and measuring outcome. Future research also needs to test the generalizability of these findings over disorders and treatment administration forms, and preferably use randomized designs to investigate whether for example a single early evaluation at week 4 with a disorder specific measure might be enough to increase the efficacy of psychotherapy.
